# In Vivo Mechanical Demands on Vertebral Body Replacements During Rehabilitation Exercises: A Multidimensional and Longitudinal Analysis

**DOI:** 10.3390/bioengineering13070753

**Published:** 2026-06-26

**Authors:** Maria Cesarina May, Andrea Zanirato, Luca Puce, Matteo Formica, Carlo Biz, Pietro Ruggieri

**Affiliations:** 1 IRCCS Azienda Ospedaliera Metropolitana, 16132 Genoa, Italy; m_may_93@web.de (M.C.M.); andrea.zanirato@unige.it (A.Z.); matteo.formica@unige.it (M.F.); 2 Department of Integrated Surgical Diagnostic Sciences (DISC), Orthopedic Clinic, University of Genoa, 16132 Genoa, Italy; 3 Department of Neuroscience, Rehabilitation, Ophthalmology, Genetics, Maternal and Child Health (DINOGMI), University of Genoa, 16132 Genoa, Italy; 4 Department of Orthopedics and Orthopedic Oncology, University of Padova, 35128 Padova, Italy; pietro.ruggieri@unipd.it

**Keywords:** vertebral body replacement, spine biomechanics, rehabilitation, implant loading, in vivo measurements, mechanical stratification

## Abstract

Background: Mechanical complications remain a concern after vertebral body replacement (VBR), especially during rehabilitation. Yet exercise prescription is often guided by body posture or single loading measures. This study characterized mechanical demands during rehabilitation exercises after VBR and examined the effects of posture and postoperative time. Methods: Telemetric in vivo load data from instrumented VBRs in the OrthoLoad database were analyzed. A total of 119 trials across 21 exercises, performed in supine, prone, seated, and standing positions, were collected from five patients over 0.1–63 postoperative months. Mechanical demand was quantified across six biomechanical domains and integrated into a composite SafetyIndex. Posture- and time-related effects were assessed using linear mixed-effects models. Worst-case demand was defined as the 95th percentile of SafetyIndex values. Results: SafetyIndex showed a right-skewed distribution (median 8.5, IQR 3.7–14.1), with marked inter-exercise variability. Composite SafetyIndex did not differ between postures (all *p* > 0.13). However, posture-dependent effects emerged at the domain level: peak shear ratio was greater in prone than in sitting, standing, and supine positions (all *p* < 0.05); peak force was greater in standing than prone (*p* = 0.007 and *p* = 0.013 in unadjusted and adjusted models); and peak resultant moment was smaller in supine than prone (*p* = 0.036 and *p* = 0.046). Postoperative time was positively associated with peak force (β = +0.40 %BW/month, *p* = 0.042), peak resultant moment (β = +0.025 Nm/month, *p* < 0.001), and SafetyIndex (β = +0.25/month, *p* = 0.011), but not peak shear ratio (*p* = 0.879). Worst-case SafetyIndex_P95 values ranged from 0.6 to 85.0, with stable ranking across percentile thresholds (Spearman’s ρ = 0.995–0.997). Conclusions: Mechanical demand after VBR is task-specific and domain-dependent and cannot be inferred from posture alone. Axial and bending-related components increased over postoperative time, whereas shear-related loading remained task-dependent.

## 1. Introduction

Severe vertebral compression fractures and neoplastic spinal lesions often require vertebral body replacement (VBR) to restore anterior column continuity, re-establish sagittal alignment, and enable early mobilization [1,2]. Despite advances in implant design and surgical technique, mechanical complications such as VBR subsidence, loss of correction, and construct failure remain clinically relevant, particularly during the early postoperative phase and in patients with poor bone quality [3,4]. These complications are not merely radiographic findings but may have important clinical consequences [5,6]. Progressive implant subsidence and loss of correction can compromise sagittal alignment, reduce foraminal height, and alter load transmission across adjacent spinal segments, potentially resulting in persistent pain, neurological deterioration, or progressive deformity [6]. Similarly, construct failure may require prolonged activity restrictions, delayed rehabilitation, or revision surgery, thereby increasing morbidity, healthcare costs, and negatively affecting patients’ quality of life [5].

Therefore, VBR should not be considered merely as an anatomical reconstruction, but rather as a biomechanical system whose stability and durability depend on the physiological loads acting on the implant during daily activities and rehabilitation [7]. For this reason, understanding the mechanical demands associated with functional tasks is essential for postoperative management and for the development of safe and effective rehabilitation protocols.

Early rehabilitation is generally encouraged after surgery to reduce deconditioning and promote functional recovery [8]. In clinical practice, however, the selection and progression of rehabilitation exercises are often guided by patient-related factors and organized according to body posture, which serves as a practical criterion for structuring postoperative rehabilitation programs [9]. From a biomechanical perspective, this approach is reductive, because the loads acting on a VBR are not determined by body weight alone, but arise from the combined effects of muscle activation, posture, lever-arm geometry, and motor control strategies.

Direct in vivo evidence supporting this concept has become available through telemeterised VBRs. In these systems, a clinically established vertebral body replacement is instrumented with integrated strain gauges, a telemetry unit, and an inductive power supply, allowing continuous in vivo recording of three-dimensional forces and moments acting on the anterior spinal column. This technology, first described by Rohlmann et al. [10], enables direct quantification of implant loading under functional conditions.

Using this approach, previous in vivo studies have shown that movements such as trunk flexion, sit-to-stand transitions, and lifting tasks can generate greater mechanical demands on the implant than relaxed standing, particularly when dynamic trunk control and long external lever arms are involved [11,12,13]. Under these conditions, implant loading reflects complex interactions between neuromuscular activation and spinal geometry rather than posture alone. Consistently, comparisons between telemetric measurements and musculoskeletal model predictions have revealed substantial discrepancies during tasks requiring complex trunk muscle coordination, suggesting that simplified modelling assumptions may underestimate the true in vivo mechanical demand acting on the implant [14,15,16].

Despite this complexity, mechanical demand has traditionally been described using unidimensional metrics, such as peak or resultant force values. However, biomechanical risk may also depend on load exposure frequency and duration, temporal stability of force application, and load directionality, including bending moments and shear components [17,18]. As a result, tasks characterized by moderate loads sustained over time or by substantial non-axial components may not be adequately captured by conventional descriptors of implant loading. Early telemetric studies have further shown that non-axial loading may increase under asymmetric or flexed configurations even in the absence of external loads [19], while pronounced inter-individual variability limits the formulation of universal recommendations based on single loading parameters [20,21].

Longitudinal in vivo investigations also suggest that implant loading patterns may change over time because of rehabilitation progression, evolving neuromuscular strategies, and structural adaptation of the treated spinal segment [22,23]. Taken together, these observations indicate that the mechanical demands acting on a VBR should be conceptualized as multidimensional and potentially time-dependent, rather than as a single scalar quantity.

The present study therefore adopted a multidimensional and longitudinal approach to the analysis of in vivo implant loading during standardized rehabilitation exercises performed in the supine, prone, seated, and standing positions. Mechanical demand was described through complementary biomechanical domains, including load intensity, cumulative exposure, temporal stability, bending moments, shear loading, and force orientation, and was synthesized into a composite indicator (SafetyIndex). The aims of the study were to characterize the distribution of mechanical demand across commonly prescribed rehabilitation exercises; determine whether such demand can be inferred from body posture alone or is better understood at the level of specific biomechanical domains; examine its evolution over postoperative time; and identify potentially critical exercises through a worst-case analysis based on high percentiles of composite mechanical demand.

## 2. Materials and Methods

### 2.1. Study Design and Data Source

This study presents an observational biomechanical analysis of in vivo loading conditions acting on VBRs during rehabilitation exercises. All data were obtained from the publicly available OrthoLoad database, which contains direct telemetric measurements of forces and moments acting on instrumented spinal implants during daily and rehabilitation-related activities [24]. Data were used exclusively for non-commercial purposes, in accordance with OrthoLoad usage policies. All analyzed trials are traceable through their original OrthoLoad file identifiers, which are reported in Supplementary Dataset S1.

### 2.2. Instrumented Implant and Load Measurement

**Figure 1:**
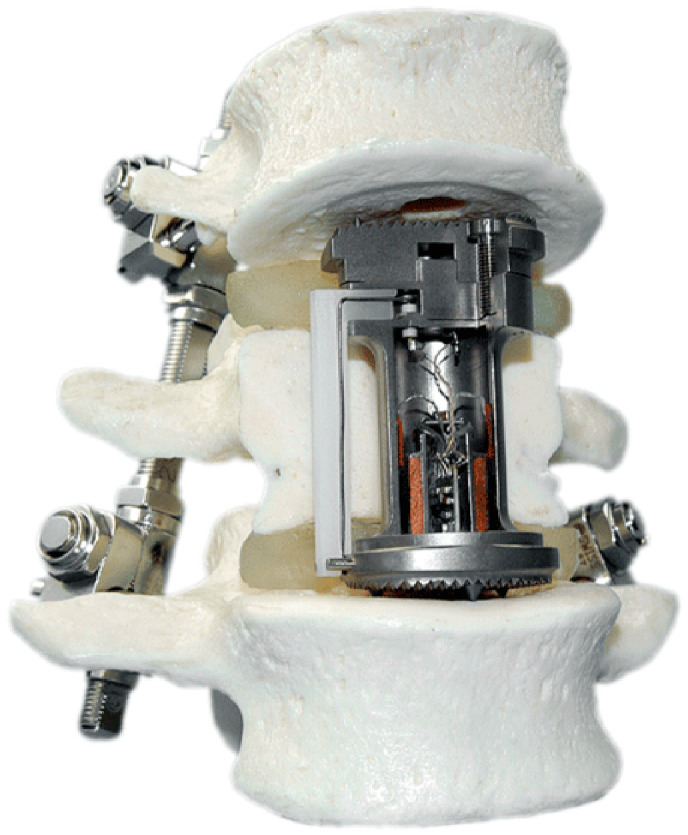
Instrumented vertebral body replacement (SYNEX) modified for in vivo measurement of spinal loads. The implant integrates six load sensors and a 9-channel telemetry transmitter housed within the implant body, enabling continuous recording of three force components and three moment components acting on the vertebral body replacement during daily activities and rehabilitation exercises. Image reproduced from OrthoLoad (Bergmann G, Damm P, eds.; Julius Wolff Institute, Berlin Institute of Health at Charité—Universitätsmedizin Berlin; 2008; http://www.OrthoLoad.com; accessed 15 December 2025 [24]), in accordance with OrthoLoad non-commercial usage policies.

In vivo loads were recorded using an instrumented VBR implant (SYNEX, Synthes), modified to allow direct measurement of three orthogonal force components and three orthogonal moment components acting on the implant. The implant integrates six load sensors and a custom-developed nine-channel telemetry system, together with an inductive power supply, enabling continuous wireless transmission of load data during functional activities. In all patients, the VBR was additionally stabilized by posterior internal fixation, as routinely applied in clinical practice. Figure 1 shows the instrumented vertebral body replacement used for direct in vivo load measurement.

### 2.3. Coordinate System and Mechanical Variables

Mechanical loads were expressed using the OrthoLoad vertebral body replacement coordinate system, defined according to the ISO 2631-1:1997 standard [25]. The coordinate axes were oriented anteriorly (x-axis), laterally to the left (y-axis), and cranially (z-axis). Forces were expressed in Newtons (N) and moments in Newton-meters (Nm). Resultant forces and resultant moments were computed as the Euclidean magnitude of the three orthogonal components.

### 2.4. Participants

Data from five patients implanted with an instrumented VBR were included in the present analysis. All patients were part of previously approved clinical studies included in the OrthoLoad database. The procedures were approved by the local ethics committee of Charité—Universitätsmedizin Berlin, and all participants provided written informed consent for the pseudonymized publication of implant load data. Four patients were male and one was female. Body mass ranged from 60 to 72 kg, and age at implantation ranged from 62 to 71 years. In four patients, the fracture and corresponding implant level were located at L1, whereas in one patient the implant was located at L3. In all cases, posterior internal fixation was used in addition to the VBR.

### 2.5. Selection of Rehabilitation Exercises

Rehabilitation-related tasks were selected from the OrthoLoad dataset [24] based on clinical relevance, availability across multiple patients, and representation of exercises performed in supine, prone, seated, and standing positions. Only tasks shared across participants and consistent with commonly adopted postoperative physiotherapy protocols were included.

### 2.6. Biomechanical Outcome Domains

Mechanical loading of the VBR was characterized using six complementary biomechanical outcome domains, each capturing a distinct aspect of implant loading derived directly from in vivo force and moment recordings. These domains were analyzed individually for domain-specific interpretation and jointly as components of a composite index of mechanical demand (SafetyIndex).

#### 2.6.1. Load Intensity

Load intensity was quantified as the peak resultant force, defined as the maximum magnitude of the resultant force recorded during each trial.

#### 2.6.2. Cumulative Exposure

Cumulative mechanical exposure was quantified as the impulse of the resultant force, calculated as the time integral of the resultant force over the entire duration of the trial.

#### 2.6.3. Stability of Force Application

Execution stability was assessed using the coefficient of variation in the resultant force time series, calculated as the ratio between the standard deviation and the mean resultant force over the trial.

#### 2.6.4. Resultant Bending Moments

Bending and torsional loading were quantified as the magnitude of the three-dimensional resultant moment vector acting on the implant.

#### 2.6.5. Shear Loading

Non-axial loading was characterized using the shear-to-compression ratio, defined as the ratio between the magnitude of the tangential (shear) force component and the compressive force component.

#### 2.6.6. Force Orientation

Force orientation was described using the obliquity angle of the resultant force vector relative to the longitudinal axis of the implant.

#### 2.6.7. Construction of the SafetyIndex

To provide an integrated representation of overall mechanical demand, a composite, dimensionless indicator named SafetyIndex was constructed. For each trial, the six biomechanical outcome domains were first normalized to a common unitless scale using min–max normalization across the entire dataset. The normalized domain values were then combined using equal weighting to compute the SafetyIndex value for each trial. Accordingly, each biomechanical domain contributed equally to the final composite index, without assigning priority to any specific loading domain. The SafetyIndex represents a multidimensional summary of mechanical demand rather than an absolute safety threshold or a direct predictor of implant failure. For interpretability, SafetyIndex values were subsequently rescaled to a 0–100 range using min–max normalization across all trials. This transformation preserves the relative ranking of trials and exercises while expressing mechanical demand on an intuitive scale. The SafetyIndex was used for descriptive visualization, posture- and time-based comparisons, and exercise-level stratification.

### 2.7. Worst-Case Mechanical Scenario Analysis

Worst-case mechanical demand for each rehabilitation exercise was quantified using the 95th percentile (P95) of SafetyIndex values across all available trials of that exercise. This percentile-based measure captures high-demand execution scenarios while limiting the influence of isolated extreme outliers. Rankings based on the 90th, 95th, and 99th percentiles were also generated to evaluate the robustness of exercise classification across different percentile thresholds.

### 2.8. Statistical Analysis

Due to non-normal data distributions, all biomechanical outcomes were summarized using median and interquartile range (IQR). Mechanical demand was characterized at three complementary levels: (i) across the entire dataset, to describe the global distribution of in vivo loading conditions; (ii) stratified by body posture (supine, prone, seated, standing), to explore posture-dependent biomechanical profiles; and (iii) at the level of individual rehabilitation exercises, to capture task-specific mechanical demand and worst-case behavior.

To evaluate associations between biomechanical outcomes, body posture, and postoperative time while accounting for repeated measurements within subjects, linear mixed-effects models were applied. Separate models were fitted for four prespecified outcomes representing complementary loading domains: SafetyIndex, peak force expressed as %BW, peak resultant moment, and peak shear ratio. For each outcome, two models were considered: a posture-only model, including posture as a fixed effect and subject as a random intercept, and an extended posture-plus-time model, in which postoperative time (months since surgery) was included as a continuous covariate.

These outcomes were selected to capture overall mechanical demand and its primary axial, bending, and non-axial components, while avoiding redundancy with correlated or derivative variables such as cumulative exposure, execution stability, and force orientation, which were retained for descriptive and composite analyses. The robustness of the worst-case exercise rankings across the P90, P95, and P99 thresholds was evaluated using Spearman rank correlation coefficients and overlap among the ten highest-ranked exercises.

Model estimates are reported as regression coefficients (β) with 95% confidence intervals (95% CI) and associated *p*-values. Inferential analyses were intended to support comparative interpretation of biomechanical patterns rather than to test causal hypotheses. All analyses were performed using Python 3.12.13 (Anaconda, Inc., Austin, TX, USA) through Jupyter Notebook 7.5.7, with custom scripts and a two-sided significance level set at *p* < 0.05.

## 3. Results

### 3.1. Study Sample and Overall Dataset Characteristics

A total of 119 trials performed by five participants were analyzed, covering 21 rehabilitation exercises executed in supine, prone, seated, and standing positions over a postoperative period ranging from 0.1 to 63 months. All trials are traceable through their original OrthoLoad file identifiers, and a complete overview of trial characteristics and biomechanical outcomes is provided in Supplementary Dataset S1.

### 3.2. Distribution of Overall Mechanical Demand

**Figure 2:**
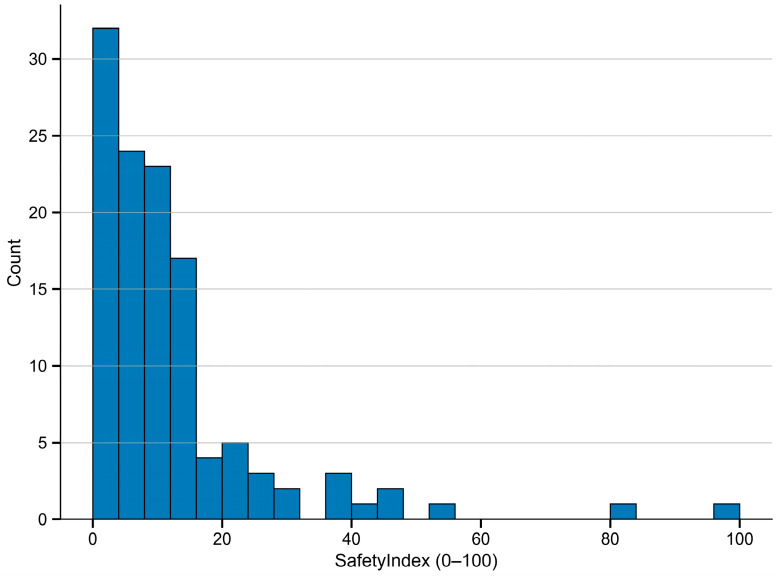
Distribution of the composite SafetyIndex (0–100) across all 119 trials. Values show a marked right-skewed distribution, with most observations clustered in the low-to-moderate range and a limited number of trials forming a pronounced upper tail.

Across the entire dataset, SafetyIndex values showed a markedly right-skewed distribution on the 0–100 scale (Figure 2). The median normalized SafetyIndex was 8.51 (IQR 3.66–14.12), with 90% of observations below 26.92 and 95% below 40.12. Only 5.0% of observations exceeded a SafetyIndex of 40, and only 2.5% exceeded 50, giving rise to a long upper tail of the distribution.

### 3.3. Changes in Mechanical Demand over Postoperative Time

**Figure 3:**
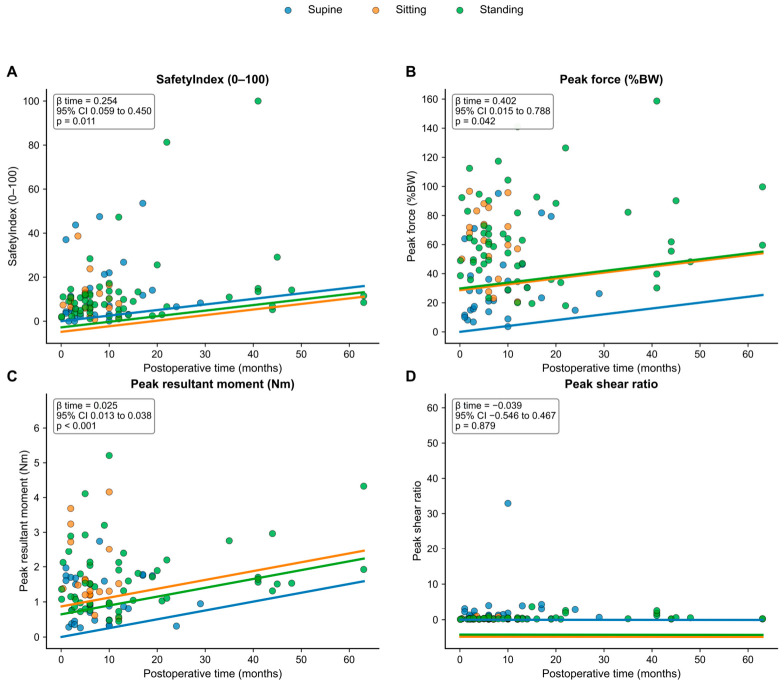
Association between postoperative time and mechanical demand outcomes. Panel (**A**) shows SafetyIndex (0–100), panel (**B**) shows peak force expressed as %BW, panel (**C**) shows peak resultant moment, and panel (**D**) shows peak shear ratio. Dots represent individual trials by posture, and lines indicate fitted values from mixed-effects models adjusted for posture.

As shown in Figure 3A, SafetyIndex values were broadly distributed throughout the postoperative period, without clustering within specific postoperative phases. Linear mixed-effects models, including posture as a fixed effect and participant as a random intercept, indicated an increase in SafetyIndex of 0.25 points per month (95% CI 0.06 to 0.45, *p* = 0.011).

Peak force expressed as %BW also increased over postoperative time (Figure 3B), with an estimated change of 0.40 %BW per month (95% CI 0.02 to 0.79, *p* = 0.042). Peak resultant moment increased by 0.025 Nm per month (95% CI 0.013 to 0.038, *p* < 0.001) (Figure 3C).

In contrast, peak shear ratio did not change significantly over postoperative time (Figure 3D; β = −0.04 per month, 95% CI −0.55 to 0.47, *p* = 0.879). Overall, postoperative time was associated with increases in the composite mechanical demand index, peak force, and peak resultant moment, whereas peak shear ratio remained unchanged throughout the postoperative period.

### 3.4. Posture-Dependent Profiles of Composite Mechanical Demand

**Figure A1:**
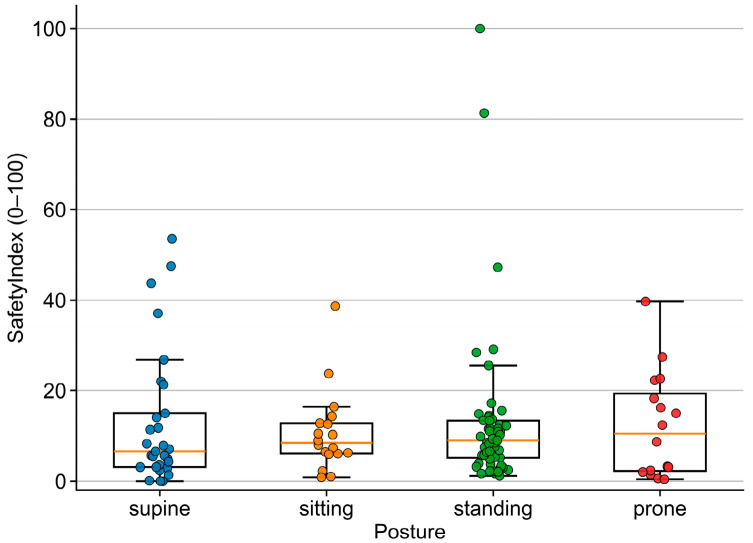
Distribution of SafetyIndex values across body postures. Boxplots with overlaid individual data points show SafetyIndex values normalized on a 0–100 scale for supine, sitting, standing, and prone exercises. Boxes represent the interquartile range, horizontal lines indicate the median, and whiskers show the data range excluding outliers.

In mixed-effects models, the composite SafetyIndex did not differ significantly between postures when prone was used as the reference condition. In the posture-only model, no significant differences were found for sitting (β = −6.50, 95% CI −15.11 to 2.10, *p* = 0.139), standing (β = −2.06, 95% CI −9.16 to 5.04, *p* = 0.569), or supine (β = −0.84, 95% CI −8.49 to 6.80, *p* = 0.829). These results were unchanged after adjustment for postoperative time. Posture-specific distributions of SafetyIndex values are shown in Figure A1.

### 3.5. Posture-Dependent Effects on Specific Biomechanical Domains

**Figure 4:**
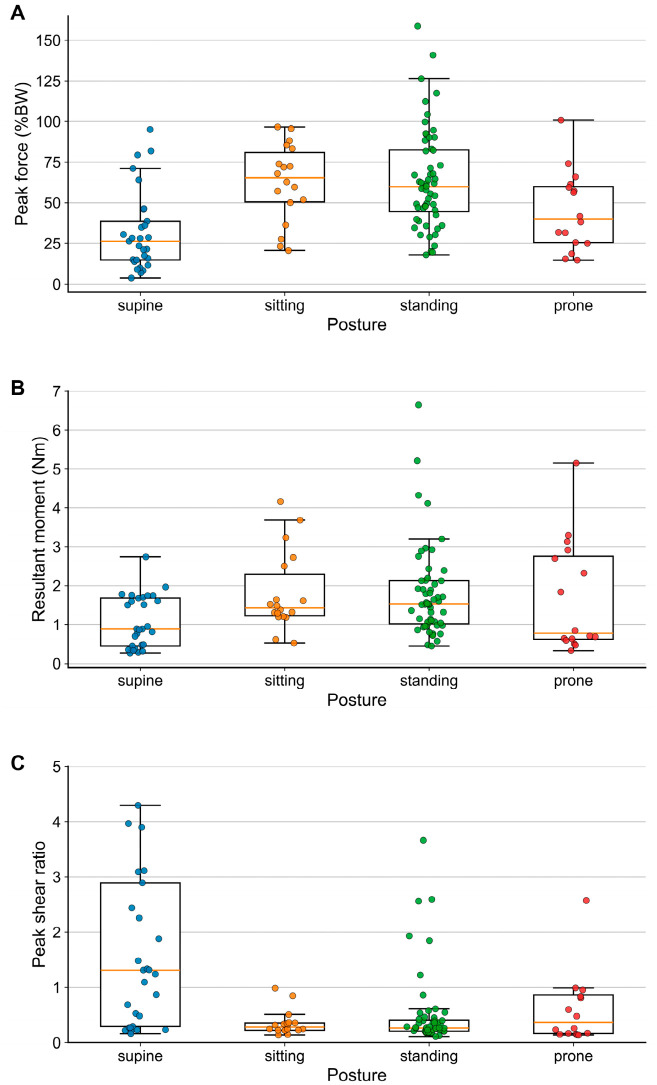
Posture-dependent distributions of selected biomechanical outcomes shown as box-and-scatter plots. (**A**) Peak force expressed as percentage of body weight (%BW), (**B**) peak resultant moment (Nm), and (**C**) peak shear ratio. Boxes represent median and interquartile range; points indicate individual trials.

Posture-dependent effects emerged when individual biomechanical domains were examined (Figure 4A–C). For peak force expressed as %BW, values were significantly greater in standing than in prone in both the posture-only model (β = +19.12 %BW, 95% CI 5.20 to 33.04, *p* = 0.007) and the posture-plus-time model (β = +17.53 %BW, 95% CI 3.76 to 31.29, *p* = 0.013). In the adjusted model, postoperative time was also significantly associated with peak force (β = +0.40 %BW per month, 95% CI 0.02 to 0.79, *p* = 0.042).

For peak resultant moment, values were significantly smaller in the supine posture than in prone in both the posture-only model (β = −0.54 Nm, 95% CI −1.04 to −0.03, *p* = 0.036) and the posture-plus-time model (β = −0.48 Nm, 95% CI −0.96 to −0.01, *p* = 0.046). Postoperative time was also positively associated with peak resultant moment (β = +0.025 Nm per month, 95% CI 0.013 to 0.038, *p* < 0.001).

The most consistent posture-dependent effect was observed for peak shear ratio (Figure 4C). Compared with prone, peak shear ratio was significantly smaller in sitting (β = −28.82, 95% CI −50.56 to −7.09, *p* = 0.009), standing (β = −25.73, 95% CI −43.66 to −7.80, *p* = 0.005), and supine (β = −20.38, 95% CI −39.69 to −1.07, *p* = 0.039). These differences remained significant after adjustment for postoperative time.

### 3.6. Exercise-Specific Worst-Case Mechanical Demand

**Figure 5:**
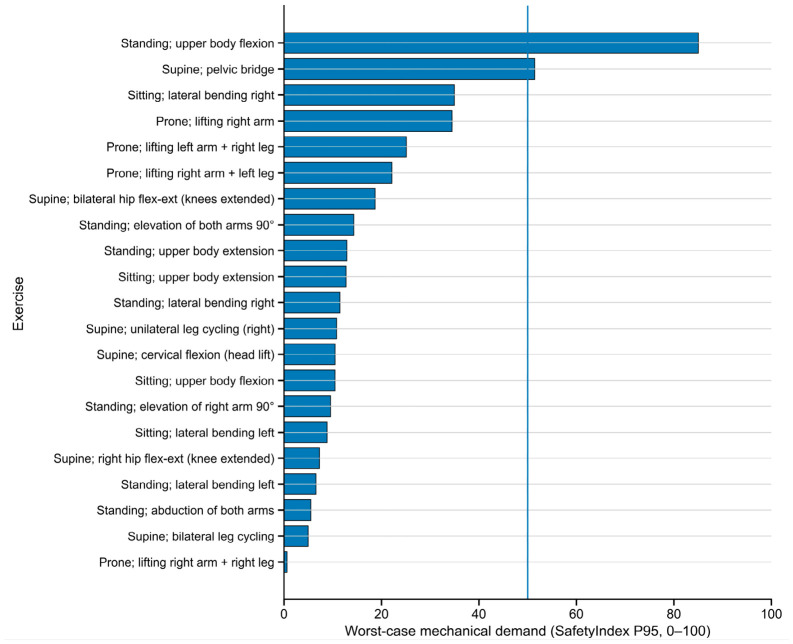
Exercise-specific worst-case mechanical demand quantified as the 95th percentile (P95) of the SafetyIndex (0–100) across all trials for each rehabilitation exercise. Exercises are ordered from highest to lowest worst-case demand.

Exercise-specific worst-case mechanical demand, defined as the 95th percentile (P95) of SafetyIndex values across all trials for each exercise, is shown in Figure 5. Worst-case SafetyIndex values varied markedly across exercises. Standing upper-body flexion showed the largest worst-case values (Safety_P95 = 85.02; Safety_P99 = 97.00; Safety_max = 100), whereas all other exercises showed Safety_P95 values ≤ 51.44.

### 3.7. Robustness of Exercise Ranking and Clinical Classification

**Figure 6:**
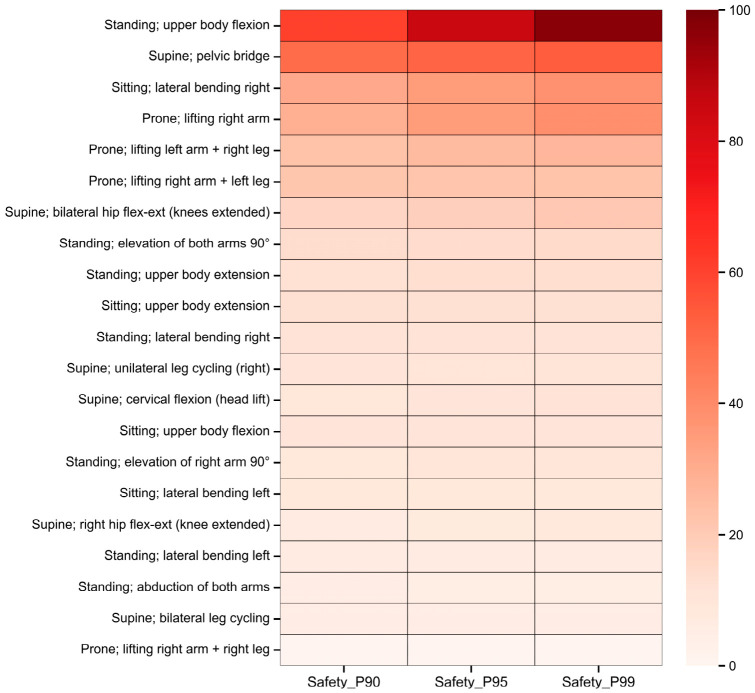
Heatmap representation of exercise-specific worst-case mechanical demand. For each rehabilitation exercise, the 90th (P90), 95th (P95), and 99th (P99) percentiles of the SafetyIndex (0–100) are shown.

**Figure A2:**
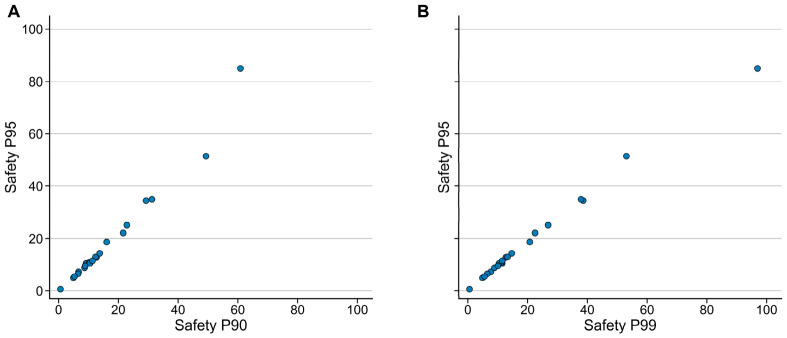
Robustness of exercise-level SafetyIndex rankings across percentile definitions. Scatter plots show the relationship between exercise-specific SafetyIndex P95 and SafetyIndex P90 (**A**) and between SafetyIndex P95 and SafetyIndex P99 (**B**). Each point represents one rehabilitation exercise, with SafetyIndex values normalized on a 0–100 scale.

The robustness of exercise ranking to the choice of percentile threshold was assessed by comparing rankings based on the 90th (P90), 95th (P95), and 99th (P99) percentiles of the SafetyIndex (Figure A2). Rankings were consistent across percentile definitions (Spearman ρ = 0.995 for P90 vs. P95 and ρ = 0.997 for P99 vs. P95), and the set of the 10 most demanding exercises was identical across all thresholds. This robustness is visually summarized in Figure 6, which shows a heatmap of P90, P95, and P99 SafetyIndex values for each exercise and illustrates the stability of worst-case mechanical demand across increasing percentile levels.

## 4. Discussion

Despite the increasing use of VBR for severe vertebral compression fractures and spinal tumors, postoperative rehabilitation remains difficult to individualize because exercise progression is often guided by practical criteria rather than by a detailed understanding of implant loading. This creates a clinically relevant need to better characterize VBR loading during rehabilitation exercises. In this context, the present study provides a multidimensional and longitudinal characterization of the mechanical demands acting on a VBR during commonly prescribed rehabilitation exercises. Overall, the findings indicate that the mechanical demand imposed on a VBR cannot be adequately inferred from body posture or from any single loading parameter in isolation, but rather from the interaction between task configuration, domain-specific biomechanical components, and execution modality.

At a global level, the distribution of SafetyIndex values was right-skewed, with most observations concentrated in the lower portion of the scale and only a limited number of observations contributing to the upper tail. This pattern suggests that, although many rehabilitation exercises are associated with modest overall mechanical demand, specific task–execution combinations may generate substantially greater mechanical demand. These findings support the need for a classification framework that goes beyond body posture alone.

Within this framework, the absence of significant posture-related differences in the composite SafetyIndex should not be interpreted as evidence that posture is mechanically irrelevant. Rather, it likely reflects the integrative nature of the index, in which biomechanical domains with different sensitivities to posture are combined into a single summary measure. When the individual domains were examined separately, clear and statistically significant posture-dependent patterns emerged. Peak force expressed as a percentage of body weight was greater in standing than in prone tasks, whereas peak resultant moment was smaller in supine than in prone conditions. The most consistent posture-dependent effect was observed for non-axial loading, with significantly greater shear ratios in prone exercises than in seated, standing, and supine tasks. Taken together, these findings highlight an important distinction: although posture alone does not reliably discriminate overall mechanical demand, it does influence specific biomechanical components that may be clinically relevant.

The present results are consistent with earlier telemetric measurements obtained from instrumented VBRs, which challenged simplified posture-based assumptions. Rohlmann et al. [11] showed that certain exercises performed in supine or seated positions, even in the absence of additional external loads, can generate implant loads comparable to those observed during relaxed standing. However, when trunk involvement becomes active under dynamic conditions, the mechanical demand increases substantially. Within the first postoperative month, trunk flexion and arm elevation performed while standing generated resultant forces of approximately 420–700 N, whereas static lying positions remained below 100 N [19]. Taken together, these observations suggest that mechanical demand is determined less by posture per se than by task-specific mechanical requirements, including external lever arms and the need for active trunk stabilization.

This principle is clearly reflected in the worst-case results of the present study. Among all analyzed exercises, standing upper-body flexion showed the greatest composite mechanical demand (Safety_P95 ≈ 85), markedly exceeding all other tasks. A plausible biomechanical explanation is that upright trunk flexion increases external lever arms, thereby amplifying bending moments, trunk muscle activation, and the forces transmitted to the implant. In line with this interpretation, previous work has shown that during trunk flexion the load–angle relationship is not necessarily monotonic: implant loads may plateau or even decrease at intermediate flexion angles, whereas the greatest peaks often occur during the return phase to the upright position [12]. This behavior underscores the relevance of movement dynamics and neuromuscular control strategies, rather than static joint angles alone, in determining spinal loading.

Previous investigations have also reported marked inter-individual variability when comparing seated and standing conditions. In particular, substantial variability in the seated-to-standing load ratio has been documented and appears to be influenced by upper-limb positioning and sagittal spinal alignment [16]. This variability is consistent with the heterogeneity observed in the present study among seated tasks. Seated exercises requiring active trunk control in the frontal plane, such as lateral bending, showed greater worst-case values than more compact seated tasks associated with reduced trunk perturbation. Likewise, prior telemetric studies have shown that strategies reducing external lever arms, such as trunk or upper-limb support, can substantially reduce implant loading [20].

The percentile-based analysis of worst-case mechanical scenarios represents a further methodological contribution of the present study. The stability of exercise ranking across different percentile thresholds (P90, P95, and P99) indicates that the identification of mechanically demanding tasks was not driven by isolated outliers, but rather reflected systematic differences in task-related mechanical demand [20,23]. This approach complements traditional analyses based on central tendency and provides a clinically meaningful framework for exercise stratification.

Regarding the temporal dimension, the composite SafetyIndex, peak force, and peak resultant moment all showed significant increases over time after surgery, whereas shear-related loading did not. This pattern suggests that rehabilitation progression may be associated primarily with increases in the axial and bending components of implant loading, while non-axial loading remains more strongly task-dependent [22]. Temporal changes in the mechanical environment, such as implant subsidence, progressive fusion, or altered segmental compliance, may also influence load transfer characteristics over time. However, these mechanisms remain speculative and cannot be directly assessed within the present dataset.

Overall, the present findings indicate that the mechanical demand acting on a VBR during rehabilitation is both task-specific and domain-specific. Although posture alone does not provide a reliable indicator of overall mechanical demand, the integration of complementary biomechanical domains allows a more informative stratification of rehabilitation exercises and supports a more rational progression strategy [15,20].

### 4.1. Clinical Implications

From a clinical perspective, the present findings support a task-oriented, rather than purely posture-based, approach to rehabilitation after VBR. Rather than classifying exercises solely according to whether they are performed in supine, prone, seated, or standing positions, clinicians should also consider observable task characteristics associated with greater mechanical demand, such as substantial trunk flexion, long upper-limb lever arms, asymmetric trunk movements, and dynamic trunk stabilization. Exercises with these characteristics may warrant closer supervision and a more gradual progression, with patient-related factors such as age, bone quality, comorbidities, symptoms, and functional recovery considered as part of the overall clinical decision-making process. The observed time-related increases in composite mechanical demand, peak force, and peak resultant moment also suggest that exercise progression should be adapted over the postoperative course, rather than assumed to be mechanically constant across recovery stages. Integrating these biomechanical considerations into rehabilitation planning may facilitate more individualized exercise progression and more informed clinical decision-making after VBR.

### 4.2. Strengths and Limitations

A major strength of the present study was the use of direct in vivo measurements obtained from an instrumented VBR, representing a reference standard for quantifying spinal implant loading. Unlike musculoskeletal modelling or indirect estimations, the OrthoLoad dataset provides telemetric recordings of forces and moments acting on the implant during real rehabilitation exercises, allowing a physiologically grounded assessment of mechanical demand. A further strength is the adoption of a multidimensional framework integrating load intensity, cumulative exposure, temporal stability, bending moments, shear loading, and force orientation, thereby capturing complementary aspects of implant loading that are rarely examined jointly in rehabilitation research.

The longitudinal nature of the dataset is an additional strength, as it allowed time-dependent changes in mechanical demand to be examined over an extended postoperative period. Moreover, the use of mixed-effects models enabled posture- and time-related effects to be assessed while accounting for repeated measurements within participants, thereby strengthening comparative analyses despite the limited number of implanted patients. Finally, the worst-case approach based on high-percentile SafetyIndex values provides a clinically relevant perspective that complements analyses based on central tendency alone.

Several limitations should also be acknowledged. First, the study was based on a small number of patients, reflecting the rarity of instrumented spinal implants and the ethical and practical constraints associated with this type of measurement. Although this limitation is partly balanced by the large number of trials and exercises analyzed, the generalizability of the findings remains limited. Second, detailed clinical information on rehabilitation protocols, symptom status, or imaging-based indicators of fusion progression was not available, preventing direct associations between mechanical demand, clinical outcomes, and structural changes over time.

In addition, the SafetyIndex should be regarded as a comparative and exploratory metric rather than an absolute indicator of implant safety or failure risk. While it preserves the relative ranking of exercises, it does not define biomechanical thresholds for safe or unsafe loading. Furthermore, although posture and postoperative time were explicitly modeled, other potentially relevant factors, such as movement velocity, muscle co-contraction, or therapist-assisted modifications, could not be quantified and may also influence implant loading.

Despite these limitations, the present study provides a comprehensive and physiologically grounded characterization of in vivo mechanical demand during rehabilitation after VBR and offers a robust biomechanical framework to support exercise selection and progression.

## 5. Conclusions

This study shows that the mechanical demand acting on a VBR during rehabilitation exercises is determined primarily by the motor task and its execution, rather than by body position alone or by isolated loading measures such as peak force. The multidimensional approach adopted here showed that exercises often considered low risk on the basis of posture alone may still generate unfavorable mechanical conditions when they involve substantial trunk activation, long lever arms, or non-axial loading. By contrast, tasks characterized by limited trunk involvement and predominantly compressive loading tended to show more stable mechanical profiles.

The use of a worst-case approach based on the 95th percentile of a composite loading index also allowed potentially critical exercises to be identified that would not have been recognized from mean values alone. Overall, these findings support the use of an in vivo, task-oriented, and multidimensional framework to guide a safer and more mechanically informed progression of rehabilitation after VBR.

## Data Availability

The data supporting the results of this study are available in Supplementary Dataset S1. This dataset includes the raw trial-level data used for the analyses, the original OrthoLoad file identifiers, exercise and posture classifications, postoperative time, and the biomechanical outcome measures derived in the study. The original in vivo load recordings are publicly available from the OrthoLoad database.

## Supplementary Materials

The following supporting information can be downloaded at https://www.mdpi.com/article/10.3390/bioengineering13070753/s1. Supplementary Dataset S1: Trial-level dataset including OrthoLoad file identifiers, task/posture classification, postoperative time, and derived biomechanical outcome measures.
